# Effects of 0.05% Cetylpyridinium Chloride Mouthwash on Halitosis and Tongue Microbiota in Patients Undergoing Orthodontic Treatment: A Double-Blind Randomized Clinical Trial

**DOI:** 10.3390/jcm14134576

**Published:** 2025-06-27

**Authors:** Natsuki Shiina, Yudai Shimpo, Kou Kikuchi, Toshiko Sekiya, Hiroshi Tomonari

**Affiliations:** Department of Orthodontics, Tsurumi University School of Dental Medicine, Yokohama 230-8501, Japan; piinurts@gmail.com (N.S.); pd23003@stu.tsurumi-u.ac.jp (K.K.); sekiya-t@tsurumi-u.ac.jp (T.S.); tomonari-h@tsurumi-u.ac.jp (H.T.)

**Keywords:** cetylpyridinium chloride, mouthwash, halitosis, volatile sulfur compounds, orthodontic treatment, multibracket appliances, tongue microbiota, 16S rRNA sequencing

## Abstract

**Background**: Halitosis is frequently observed in patients undergoing orthodontic treatment with multibracket appliances, primarily due to volatile sulfur compounds (VSCs) produced by oral anaerobic bacteria. Cetylpyridinium chloride (CPC) is a widely used antimicrobial agent in oral care products and may help alleviate halitosis.This study aimed to evaluate the effects of 0.05% CPC mouthwash on halitosis, oral hygiene indices, and the tongue microbiota in orthodontic patients with elevated VSC levels. **Methods**: In this randomized, double-blind, placebo-controlled clinical trial, 30 orthodontic patients with elevated VSCs (≥150 ppb) were assigned to a CPC mouthwash group or a placebo group. Participants used the assigned mouthwash three times daily for 1 month. Halitosis was quantitatively assessed by gas chromatography (Oral Chroma™), and oral hygiene parameters including Plaque Index (PI), Gingival Index (GI), Tongue Coating Index (TCI), and unstimulated salivary flow rate were evaluated at baseline and after the intervention. The tongue microbiota was analyzed by 16S rRNA sequencing. **Results**: The CPC mouthwash group showed significant reductions in total VSCs, hydrogen sulfide, methyl mercaptan, PI, GI, and TCI (*p* < 0.05), while salivary flow rate and dimethyl sulfide remained unchanged. Microbiome analysis revealed decreases in halitosis-associated genera (*Actinomyces*, *Corynebacterium*, *Tannerella*) and increases in beneficial species such as *Streptococcus salivarius*. **Conclusions**: CPC mouthwash (0.05%) effectively reduced halitosis and improved oral hygiene parameters in orthodontic patients, likely through modulation of the tongue microbiota. This mouthwash may serve as a safe and practical adjunct to conventional oral hygiene practices during orthodontic treatment.

## 1. Introduction

Halitosis, or bad breath, is prevalent condition affecting a significant portion of the population and is particularly pronounced among individuals undergoing orthodontic treatment with multibracket appliances [1,2]. This condition is primarily associated with the production of volatile sulfur compounds (VSCs), of which the three most common are hydrogen sulfide (H_2_S), methyl mercaptan (CH_3_SH), and dimethyl sulfide ((CH_3_)_2_S) by anaerobic bacteria that colonize the tongue dorsum and periodontal pockets [3,4,5].

Patients with fixed orthodontic appliances often face challenges in maintaining proper oral hygiene, leading to increased plaque accumulation, gingival inflammation, and tongue coating, all of which are key contributing factors in the development of halitosis [6,7,8]. In addition to objective markers, self-perceived halitosis is also commonly reported among orthodontic patients [9]. Effective management of halitosis in those individuals is crucial for improving quality of life and satisfaction with orthodontic treatment [10].

Cetylpyridinium chloride (CPC) is a quaternary ammonium compound with broad-spectrum antimicrobial activity. It is commonly incorporated into oral hygiene products due to its ability to reduce plaque formation, gingival inflammation, and oral malodor [11,12,13]. Several clinical trials have demonstrated the efficacy of CPC in improving clinical indices of oral health and modulating the oral microbiota toward a health-associated profile [14,15,16,17]. Systematic reviews and meta-analyses have further underscored the benefits of CPC-containing formulations in managing gingivitis and reducing cariogenic biofilms [18,19].

Moreover, comparative studies have shown that CPC-containing mouthwashes significantly reduce VSC levels and suppress tongue-residing malodor-producing bacteria, indicating their relevance in halitosis management among orthodontic patients [20]. Other studies have noted strong correlations between halitosis and periodontal pathogens identified within the tongue coating, reinforcing the need for interventions targeting these sites [21,22]. In parallel, novel oral biomarkers have been proposed for halitosis diagnosis and monitoring, including microbial signatures in the tongue microbiota [23].

Recent microbiome research has highlighted the critical role of tongue coating in the pathogenesis of halitosis and its association with specific VSC-producing genera such as *Fusobacterium*, *Prevotella*, and *Porphyromonas* [24,25,26,27]. As the tongue dorsum harbors a diverse and stable microbial community closely linked to oral malodor, non-invasive sampling techniques targeting this area have become increasingly important in microbiome-based halitosis research [28]. The advent of 16S rRNA gene sequencing has enabled more detailed insights into the microbial composition and dynamics of the tongue dorsum, supporting the development of personalized, microbiome-informed strategies for halitosis management [29,30,31]. CPC’s potential to selectively modulate malodor-associated microbial communities while preserving overall microbial diversity is of increasing research interest [32,33,34].

However, to date, no studies have comprehensively evaluated both clinical outcomes and tongue microbiota changes induced by CPC mouthwash in orthodontic patients with halitosis using a combination of quantitative VSC measurement and 16S rRNA gene sequencing. This study represents a novel approach integrating clinical and microbiome analyses in this specific patient population.

The present study aims to evaluate the clinical and microbiological effects of a 0.05% CPC-containing mouthwash in orthodontic patients with halitosis through a randomized, double-blind, placebo-controlled clinical trial. The primary objective is to assess changes in VSC levels, with secondary outcomes including the Plaque Index (PI), Gingival Index (GI), Tongue Coating Index (TCI), unstimulated salivary flow rate, and microbial composition of the tongue dorsum as determined by 16S rRNA gene analysis [20,35]. Given that reduced unstimulated salivary flow has been shown to be significantly associated with both the presence and severity of halitosis and identified as a predictive factor for strong halitosis in previous research [36], salivary flow rate was included as one of the secondary outcomes in the present study.

## 2. Materials and Methods

### 2.1. Trial Design

This study was designed as a randomized, double-blind, placebo-controlled, superiority trial, with two parallel groups at the Department of Orthodontics, Tsurumi University Dental Hospital. It was conducted to evaluate changes in halitosis and oral microbiome when using mouthwash in patients with halitosis undergoing orthodontic treatment using multibracket appliances. The follow-up period was 1 month. The primary outcome, halitosis, was assessed by measuring VSCs using gas chromatography (Oral Chroma™, NISSHA FIS Inc., Osaka, Japan). Gas chromatography is a validated method for quantifying concentrations of the three VSCs separately: hydrogen sulfide (H_2_S), methyl mercaptan (CH_3_SH), and dimethyl sulfide ((CH_3_)_2_S) [1]. The patient exhalation sample is introduced into the device using a syringe, and the compounds are identified and quantified using a computer-based database [2]. According to the literature, a baseline total VSC level of ≥150 ppb (parts per billion) is indicative of halitosis [3]. Secondary outcomes, including clinical periodontal indices and oral microbiota composition, were also assessed. No methodological changes were made after the trial commenced.

### 2.2. Participants, Eligibility Criteria, and Setting

The study was conducted in patients undergoing orthodontic treatment with multibracket appliances at the Department of Orthodontics, Tsurumi University Dental Hospital. Inclusion criteria were (1) age 16–40 years; (2) use of fixed conventional labial multibracket appliances on both arches; (3) appliances bonded to at least 24 teeth for ≥4 months; (4) expected treatment duration ≥1 month; (5) for extraction cases, ≥2 months post-extraction; (6) good general health; and (7) initial total VSCs ≥150 ppb [3]. Inclusion criteria required that multibracket appliances had been bonded for at least 4 months prior to enrollment. At the time of baseline measurement, all participants had been undergoing orthodontic treatment for between 6 months and 1 year 5 months. Exclusion criteria included active caries, periodontitis (pockets ≥4 mm), enamel/dentin anomalies, craniofacial syndromes, psychological conditions, tobacco use [23], recent antibiotics (<2 months), recent mouthwash use (<3 weeks), CPC allergy, or concurrent study participation [8].

From November 2024 to January 2025, 35 orthodontic patients were screened. After eligibility assessments, four were excluded and one declined participation. A total of 30 participants (12 males, 18 females; mean age 25 ± 5.1 years) were randomly assigned (*n* = 15 each group) and completed the study. All measurements were performed by a single examiner at the Department of Orthodontics.

### 2.3. Sample Size Calculation

Sample size was calculated using G*Power (Universitat Kiel, Kiel, Germany), based on a prior study reporting a mean expected VSC difference of 50 ppb, SD = 40, α = 0.05, and power = 80% [3]. The required sample size was 12 per group, increased to 15/group (30 total) to account for potential dropout.

### 2.4. Randomization

Participants were randomized using a coin-toss method supported by computer-generated random numbers. Numbers were classified as “heads” (CPC group) or “tails” (placebo group). Allocation data were anonymized and stored securely offline in a locked office at Department of Orthodontics, Tsurumi University Dental Hospital, Kanagawa, Japan. Figure S1 shows a flowchart of the patients’ allocations and dropouts based on the Consolidated Standards of Reporting Trials (CONSORT) [37].

### 2.5. Blinding

Mouthwash bottles were identically packaged to blind both participants and examiners. Allocation was performed by a third party not involved in the intervention or evaluation. This individual was responsible for labeling and distributing the mouthwash solutions based on the randomization protocol.

### 2.6. Intervention

The CPC mouthwash group received a 0.05% CPC-containing mouthwash (Mondamin HABIT PRO^®^, Earth Corporation, Tokyo, Japan). Active ingredients included CPC, tranexamic acid (TXA), and dipotassium glycyrrhizinate (GK_2_). Inactive ingredients were glycerin, PG, xylitol, peppermint flavor, PEG-hydrogenated castor oil, phosphate buffers, parabens, and Green No. 201. Participants were instructed to rinse with 10 mL of the assigned mouthwash for 30 s three times daily after toothbrushing for 1 month. No additional oral hygiene instruction was provided immediately prior to the intervention, as the study was designed to assess the effects of the mouthwash itself without the influence of concurrent hygiene education.

The placebo mouthwash group used a formulation identical in appearance, flavor, and packaging but without the active ingredients. Both groups received identical instructions. Double-blinding was maintained throughout the study. Participants’ mouthwash use compliance was confirmed by filling out a checklist.

### 2.7. Outcome Variables

#### 2.7.1. Assessment of Halitosis

Halitosis was the primary outcome, assessed by measuring VSCs (H_2_S, CH_3_SH, ((CH_3_)_2_S) using Oral Chroma™. Assessments were performed at baseline (T0) and after the 1-month intervention period (T1). Total VSCs were also calculated as the sum of the three compounds.

Measurements were carried under the following standardized conditions: (1) at least 2 h had elapsed after toothbrushing, (2) participants were instructed to refrain from eating or drinking (except for water) prior to testing, (3) intake of odor-inducing foods (e.g., garlic, spices) was prohibited within 24 h before the measurement, (4) no mouthwash use was allowed on the day of evaluation.

The sampling procedure was as follows: (1) a disposable plastic syringe (1 mL) was used to collect the oral air sample, (2) participants were instructed to close their mouths and breathe nasally for 30 s before sampling, (3) a gas sample was obtained by inserting the syringe into the oral cavity and pulling the plunger, (4) the syringe hub was then inserted into the inlet of the Oral Chroma™ device, and 0.5 mL of the sample air was injected for analysis. This protocol enables reliable and reproducible quantification of intraoral VSCs and has been widely adopted in halitosis research [38].

#### 2.7.2. Assessment of Oral Hygiene Status

As indicators of oral hygiene status, PI-M and GI were evaluated per Silness and Löe’s methods [39] at baseline (T0) and after the 1-month intervention period (T1).

For the PI-M, each tooth surface was divided into mesial, distal, and incisal areas relative to the orthodontic bracket. Plaque accumulation in each area was graded using a four-point scale: 0: no plaque, 1: thin plaque detectable only after applying a disclosing agent or probing, 2: moderate plaque visible to the naked eye at the gingival margin, 3: heavy plaque accumulation covering the gingival margin and adjacent tooth surfaces. The average score for each tooth was calculated, and the total PI-M score for each participant was obtained by summing the scores of all examined teeth. Given that a maximum of 28 teeth were evaluated, and each tooth could score up to 3 points, the highest possible total PI-M score was 84.

The GI was assessed by examining three buccal areas (mesial, central, and distal) of each tooth, including banded molars. The inflammation was scored using the following criteria: 0: normal gingiva, 1: mild inflammation with slight color and texture changes and no bleeding on probing, 2: moderate inflammation with redness, swelling, and bleeding on probing, 3: severe inflammation with marked swelling, ulceration, and spontaneous bleeding. The average score per tooth was calculated, and then the total GI score for each participant was derived by summing the scores from all teeth. As with PI-M, the maximum possible GI score was 84 when all 28 teeth were assessed. These indices have been validated for use in orthodontic populations and provide reliable measures of oral hygiene and gingival health [40].

#### 2.7.3. Assessment of Tongue Coating Index (TCI)

The dorsal surface of the tongue was visually assessed and divided into nine equal sections (three anterior, three middle, and three posterior) at baseline (T0) and after the 1-month intervention period (T1). Each section was scored based on the amount of tongue coating observed, using the following scale: score 0 = no visible coating; score 1 = thin coating partially covering the area; score 2 = thick coating extensively covering the area. The total TCI score was calculated by summing the scores of all nine sections, resulting in a maximum possible value of 18.

In this study, the final TCI value was obtained by dividing the total score by 18, yielding a standardized index ranging from 0 to 1. In accordance with the diagnostic criteria for oral hypofunction, a TCI value of 0.5 or below is considered indicative of poor oral hygiene status [39]. This method has been validated in prior studies and is widely used for clinical assessment of tongue hygiene [41].

#### 2.7.4. Unstimulated Salivary Flow Rate

The unstimulated salivary flow rate was measured by the Watt method [42] at baseline (T0) and after the 1-month intervention period (T1). Participants refrained from eating, drinking, or performing oral care for ≥90 min. A pre-weighed cotton roll was placed sublingually for 5 min.The difference in weight before and after saliva collection was used to calculate the flow rate (mL/min).

#### 2.7.5. Microbial Sampling Procedure

To analyze the oral microbiota, tongue dorsum swab samples were collected from each participant at baseline (T0) and after the 1-month intervention period (T1). The sampling was conducted according to a previously established protocol [28], using a sterile cotton swab gently placed on the mid-dorsal surface of the tongue and left in place for 20 s without applying pressure. The biological material that adhered to the swab was then used for DNA extraction and subsequent microbiome analysis.

### 2.8. Microbiome Analysis

Tongue swabs were collected, suspended in 4 M guanidine thiocyanate, and subjected to mechanical lysis. DNA was extracted using a Maxwell RSC Blood DNA Kit (Promega, Madison, WI, USA). The V1-V12 regions of the 16S rRNA gene were amplified and sequenced using an Ion S5^TM^ platform (Thermo Fisher Scientific, Waltham, MA, USA). Taxonomic classification was performed using Ion Reporter Software (ver. 5.18, Thermo Fisher Scientific, Waltham, MA, USA) and the validated reference databases “Green Genes” (publicly available) and “Micro SEQ” (Thermo Fisher Scientific). Microbiome analysis was conducted at SheepMedical Inc. (Kanagawa, Japan).

### 2.9. Statistical Analysis

Data were analyzed using SPSS version 27.0 (IBM Japan, Tokyo, Japan). Normality of distribution was assessed using the Shapiro–Wilk test (Table S1). Based on these results, most clinical parameters did not follow a normal distribution. Therefore, data are presented as median (interquartile range) for clinical parameters and as mean ± standard deviation for age. Between-group comparisons were performed using the Mann–Whitney U test for clinical parameters, the independent-samples *t*-test for age, and the Chi-square test for sex distribution. Within-group comparisons of clinical parameters were performed using the Wilcoxon signed-rank test. Descriptive statistics were used for microbiome data. A *p*-value of <0.05 was considered statistically significant.

### 2.10. Ethics

Ethical approval for this study was obtained from the Ethics Committee of Tsurumi University School of Dental Medical (Approval Number: 124008, 4 November 2024), and this study was registered with the UMIN Clinical Trials Registry (UMIN ID: 000055502, 17 September 2024). Written informed consent was obtained from all participants. Consent was obtained from those over 18 years of age directly and from the parents of those under 18 years of age.

The mouthwash product Mondamin HABITPRO^®^ (classified as a quasi-drug under the Japanese Pharmaceutical and Medical Device Act) and the placebo mouthwash used in this study were provided free of charge by Earth Corporation Pro Dental Relation Dept (Tokyo, Japan). Prior to the start of the study, the conflict of interest was reviewed and approved by the Conflict of Interest Committee of the School of Dental Medicine, Tsurumi University (Approval No. 224004). No financial support, materials, equipment, personnel, or honoraria were received for this study beyond provision of the mouthwash products.

## 3. Results

### 3.1. Baseline Characteristics (T0) of the Participants in This Study

The baseline demographic and clinical parameters of the CPC mouthwash group and the placebo group are presented in Table 1. There were no statistically significant differences between the two groups in terms of age, gender distribution, or baseline values for any clinical parameters, including VSCs, PI, GI, TCI, and unstimulated salivary flow (all *p* > 0.05).

### 3.2. Changes in VSC Levels by Participant

**Figure 1:**
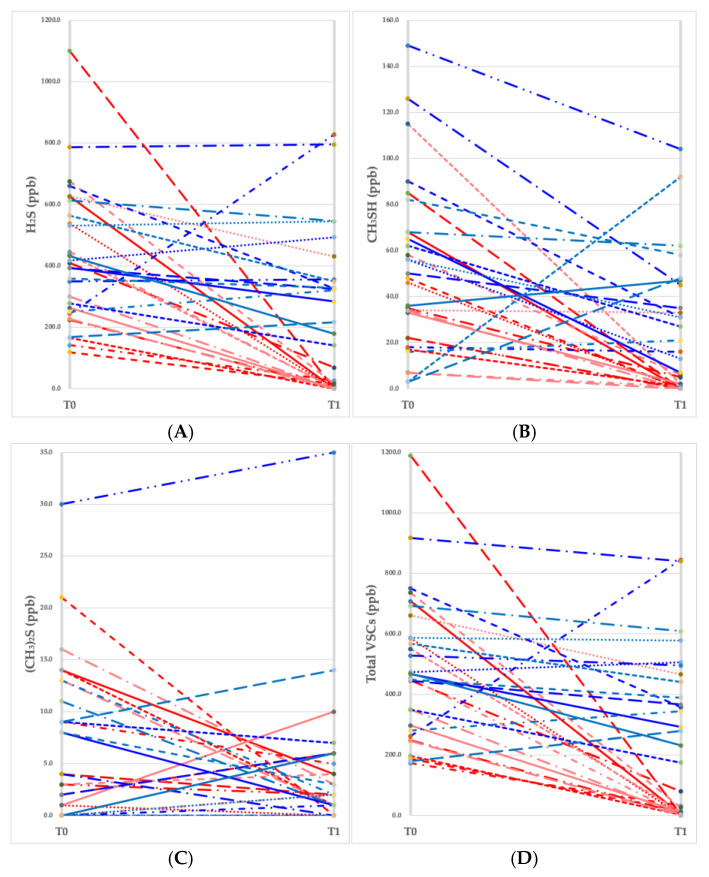
Changes in the values of volatile sulfur compounds (VSCs) measured at baseline (T0) and a month later (T1). (**A**) Hydrogen sulfide (H2S), (**B**) methyl mercaptan (CH3SH), (**C**) dimethyl sulfide ((CH3)2S), (**D**) total VSC level. Each red line represents an individual participant (*n* = 15) in the CPC mouthwash group, and each blue line represents an individual participant (*n* = 15) in the placebo mouthwash group.

Individual trajectories of VSC values (H_2_S, CH_3_SH, (CH_3_)_2_S, and total VSCs) were monitored for each participant at baseline (T0) and after 1 month (T1). As illustrated in Figure 1A–D, the CPC mouthwash group showed a remarkable reduction in hydrogen sulfide and methyl mercaptan levels, along with a substantial decline in total VSC values, indicating a significant decrease in halitosis.

### 3.3. Within-Group Comparisons

In the CPC mouthwash group, statistically significant reductions were observed in H_2_S (*p* = 0.001), CH_3_SH (*p* = 0.001), total VSCs (*p* = 0.001), PI (*p* = 0.001), GI (*p* = 0.012), and TCI (*p* = 0.003) from T0 to T1. No significant change was observed in unstimulated salivary flow (*p* = 0.107) (Table 2).

In the placebo mouthwash group, only PI showed a significant reduction after 1 month (*p* = 0.004). Other parameters, including VSCs, GI, TCI, and salivary flow, did not differ significantly from baseline (all *p* > 0.05) (Table 2).

### 3.4. Between-Group Comparisons

At T1, the CPC mouthwash group exhibited significantly lower levels of hydrogen sulfide (*p* < 0.001), methyl mercaptan (*p* < 0.001), total VSCs (*p* < 0.001), PI (*p* = 0.026), GI (*p* = 0.004), and TCI (*p* < 0.001) compared to the placebo group. No significant difference was observed for unstimulated salivary flow (*p* = 0.595). These results are summarized in Table 1.

### 3.5. Microbiome Results

#### 3.5.1. Sequence Data

A total of 60 tongue dorsum samples collected from 30 participants (before and after the intervention) were subjected to 16S rRNA gene sequencing. After quality control, all samples were retained, with read counts ranging from 23,367 to 133,275 per sample. From these sequences, bacterial taxa were clustered and classified into 10 phyla, 20 classes, 37 orders, 69 families, 137 genera, and 333 species.

#### 3.5.2. Oral Microbiome Structure

**Figure 2:**
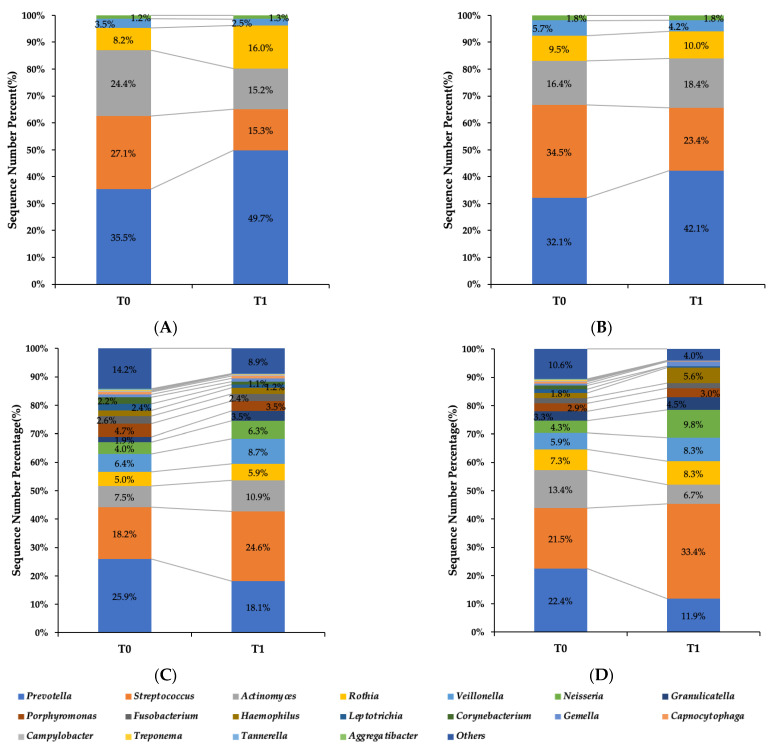
Oral microbiome structure before and after intervention. Data are presented separately at the phylum and genus levels. (**A**) The CPC mouthwash group at the phylum level; (**B**) the placebo mouthwash group at the phylum level; (**C**) the CPC mouthwash group at the genus level; (**D**) the placebo mouthwash group at the genus level.

At the phylum level, the analysis revealed notable changes following the intervention. In the CPC mouthwash group, the relative abundance of *Firmicutes* increased, while that of *Actinobacteria* and *Bacteroidetes* decreased. In contrast, the placebo group showed no marked changes in the dominant phyla over time (Figure 2A,B).

At the genus level, a decline in the relative abundance of several VSC-producing bacteria, including *Actinomyces*, *Corynebacterium*, and *Tannerella*, was observed in the CPC group. In contrast, beneficial or neutral genera such as *Rothia* and *Streptococcus* remained relatively stable throughout the study period (Figure 2A,B). The placebo group did not exhibit comparable shifts in genus composition.

These microbial shifts are consistent with a reduction in halitosis-associated taxa and support the antimicrobial effect of CPC, particularly in modulating the tongue-coating microbiota.

#### 3.5.3. Species-Level Changes in Oral Microbiota

Among the 333 species detected, only those showing a relative abundance change of ≥0.1% between baseline (T0) and post-intervention (T1) were included in the comparative analysis. Table 3 presents these species-level changes for both the CPC and the placebo groups.

In the CPC mouthwash group, notable reductions were observed in several anaerobic bacteria known to be associated with halitosis and oral infections, including *Prevotella melaninogenica* (−4.78%), *Actinomyces sp.* (−1.64%), *Prevotella histicola* (−1.55%), *Actinomyces gerencseriae* (−1.34%), and *Corynebacterium matruchotii* (−1.15%). On the other hand, species such as *Streptococcus salivarius* (+4.51%), *Streptococcus infantis* (+5.89%), *Neisseria flavescens* (+3.19%), and *Haemophilus parainfluenzae* (+3.50%) showed increased abundance, many of which are considered part of the healthy commensal flora.

In the placebo mouthwash group, while minor fluctuations were observed, they were neither as extensive nor as directional. For instance, *Prevotella copri* (−2.43%) and *Streptococcus anginosus* (−1.78%) showed declines, while *Streptococcus salivarius* (+5.23%) and *Veillonella parvula* (+2.50%) increased slightly. However, these changes did not appear to represent a consistent trend towards oral health improvement.

These results suggest that the CPC mouthwash contributed to a targeted reduction of malodor-associated species and a relative increase in beneficial commensals, reflecting a potential rebalancing effect on the tongue coating microbiome.

#### 3.5.4. Harm

No harm was observed from the use of the mouthwash.

## 4. Discussion

This randomized, double-blind, placebo-controlled clinical trial demonstrated that a 0.05% CPC mouthwash significantly reduced halitosis and improved oral hygiene in orthodontic patients with multibracket appliances. The reduction in VSCs, particularly hydrogen sulfide and methyl mercaptan, is consistent with previous findings that attribute CPC’s efficacy to its antimicrobial effects on anaerobic gram-negative bacteria responsible for oral malodor [8,11,20].

Improvements in clinical indices, including PI, GI, and TCI, suggest that CPC not only addresses halitosis, but also improves periodontal and tongue hygiene. This aligns with prior evidence that CPC inhibits plaque formation and gingival inflammation [14,15,18]. The significant reduction in tongue coating is particularly relevant, as the dorsum of the tongue serves as a major reservoir for VSC-producing bacteria [24,33].

Microbiome analysis further supported these findings. The CPC mouthwash group exhibited reductions in key halitosis-associated taxa such as *Actinomyces*, *Corynebacterium*, and *Tannerella*, while beneficial species like *Streptococcus salivarius* and *Neisseria flavescens* increased. In comparison with previous research, our findings indicate a relatively stronger improvement in halitosis-related parameters. Tsironi et al. (2023) evaluated the use of a mastic-based mouthwash in orthodontic patients and reported significant reductions in halitosis and plaque indices; however, changes in gingival inflammation were modest and less consistent [38]. Similarly, Herrera et al. (2018) conducted a 3-month study using a CPC-containing dentifrice and mouthwash in adolescents with fixed appliances, observing moderate improvements in plaque and gingival scores, but with greater interindividual variability [43]. Compared to these studies, our trial demonstrated clearer and more uniform improvements across VSC levels, clinical indices, and microbiota composition. These differences may be attributable to stricter inclusion criteria, higher baseline VSC levels, or the specific formulation and usage protocol of the 0.05% CPC mouthwash employed in this study.

Unstimulated salivary flow rate did not significantly change post-intervention, which is consistent with prior reports indicating that CPC mouthwash does not adversely affect salivary gland function [6]. However, the role of saliva in halitosis is nonetheless important, as lower resting flow rates have been associated with stronger malodor in previous studies [36].

Interestingly, dimethyl sulfide (CH_3_)_2_S levels did not show significant reductions in the CPC mouthwash group, in contrast to the substantial decreases observed for hydrogen sulfide (H_2_S) and methyl mercaptan (CH_3_SH). This discrepancy may be explained by the different origins and physicochemical properties of these volatile sulfur compounds. While H_2_S and CH_3_SH are primarily produced by anaerobic proteolytic bacteria localized in the tongue dorsum and periodontal pockets, dimethyl sulfide is more commonly associated with systemic or extraoral sources, including respiratory tract and gastrointestinal conditions [1,3,20]. Furthermore, dimethyl sulfide is more volatile and lipid-soluble, allowing it to diffuse into the bloodstream and be excreted through the lungs, contributing to halitosis of systemic origin. Prior studies have reported that oral antiseptics such as CPC or chlorhexidine show limited effectiveness against dimethyl sulfide [9,11,19]. These findings indicate that cases of elevated dimethyl sulfide may require broader diagnostic approaches beyond intraoral intervention. While chlorhexidine is widely recognized for its strong antimicrobial properties and is frequently used in clinical settings, it was not included as a control in this study due to potential adverse effects such as tooth staining and taste disturbance, which may have affected patient compliance in a short-term trial.

Importantly, the results of this study support the clinical value of incorporating CPC mouthwash into comprehensive oral hygiene protocols for orthodontic patients. While traditional strategies have focused on mechanical cleaning, such as toothbrushing and tongue scraping, chemical adjuncts like antimicrobial mouthwashes offer complementary benefits by targeting areas that are difficult to reach by physical means.

Moreover, the findings suggest that establishing interdisciplinary oral hygiene management plans that include the use of mouthwash may contribute significantly to halitosis control during orthodontic treatment. Collaboration among dental professionals—orthodontists, general dentists, and dental hygienists—is essential in developing and implementing standardized care pathways tailored to the specific hygiene challenges of orthodontic appliances. The creation and dissemination of such shared protocols may improve not only clinical outcomes but also patient adherence and satisfaction throughout treatment.

This study has several limitations. First, although the sample size was statistically powered, it remains relatively small and limited to a single geographic location. Combined with the short observation period of 1 month, these factors may limit the generalizability of the finding. The relatively short observation period of 1 month was chosen to minimize participant burden and ensure adherence. However, it may not fully capture the long-term sustainability of CPC’s effects. Future studies with extended follow-up are needed to validate the durability of these findings. Second, the participants were relatively healthy individuals undergoing routine orthodontic care, which may not reflect populations with comorbidities or active periodontal disease. This selection bias should be taken into account when interpreting the results and considering the applicability to broader clinical populations. Furthermore, while 16S rRNA sequencing provides valuable taxonomic insights, a limitation of our microbiome analysis is that 16S rRNA sequencing provides taxonomic, but not functional, insights into microbial activity. Future studies incorporating metagenomic or metabolomic approaches would be valuable to elucidate microbial interactions and VSC metabolic pathways. Such approaches could help clarify not only compositional, but also mechanistic, changes associated with CPC use.

## 5. Conclusions

In conclusion, 0.05% CPC mouthwash effectively reduces halitosis in orthodontic patients by lowering VSC concentrations and improving oral hygiene parameters such as plaque, gingival inflammation, and tongue coating. Moreover, CPC favorably modulates the tongue microbiota, reducing pathogenic taxa while preserving commensal species.

These findings support the use of CPC-containing mouthwash as a safe, targeted, and effective adjunctive strategy for managing halitosis and maintaining oral health in individuals undergoing orthodontic treatment.

## Figures and Tables

**Table 1:** Comparison of clinical parameters between the CPC and placebo mouthwash groups at T0 and T1.

Parameter	Time Point	CPC Group (*n* = 15)	Placebo Group (*n* = 15)	*p*-Value
Age (years)	T0	24.5 ± 5.5	25.5 ± 4.8	0.597
Sex (male/female)	T0	6/9	6/9	1.000
H_2_S (ppb)	T0	409.00 (225.50–582.00)	394.00 (313.50–547.50)	0.713
	T1	1.00 (0.00–15.50)	328.00 (302.50–519.00)	<0.001 **
CH_3_SH (ppb)	T0	34.00 (22.00–53.00)	56.00 (27.00–75.00)	0.250
	T1	0.00 (0.00–2.00)	35.00 (24.00–53.00)	<0.001 **
(CH_3_)_2_S (ppb)	T0	3.00 (1.00–13.50)	4.00 (0.00–9.00)	0.486
	T1	2.00 (1.50–4.50)	2.00 (0.50–6.00)	0.713
Total VSCs (ppb)	T0	446.00 (248.00–623.00)	468.00 (397.00–577.00)	0.512
	T1	6.00 (2.50–20.50)	389.00 (318.00–542.00)	<0.001 **
PI	T0	16.00 (7.55–18.25)	11.30 (7.75–16.65)	0.624
	T1	5.50 (2.90–6.75)	7.00 (5.90–9.40)	0.026 *
GI	T0	9.00 (4.50–10.50)	6.80 (4.25–14.15)	0.902
	T1	3.00 (2.40–6.40)	5.30 (4.25–7.05)	0.081
TCI	T0	0.50 (0.30–0.70)	0.60 (0.45–0.85)	0.202
	T1	0.20 (0.20–0.30)	0.60 (0.50–0.65)	<0.001 **
Unstimulated Saliva (mL)	T0	0.20 (0.20–0.40)	0.20 (0.15–0.55)	0.902
	T1	0.40 (0.25–0.50)	0.30 (0.15–0.70)	0.595

Abbreviations: H_2_S = hydrogen sulfide; CH_3_SH = methyl mercaptan; (CH_3_)_2_S = dimethyl sulfide; VSCs = volatile sulfur compounds; PI = Plaque Index; GI = Gingival Index; TCI = Tongue Coating Index. Statistical significance: *p* < 0.05 (*), *p* < 0.01 (**).

**Table 2:** Within-group comparisons of clinical parameters from T0 to T1.

Parameter	Group	T0 Median (IQR)	T1 Median (IQR)	*p*-Value
H_2_S (ppb)	CPC	409.00 (225.50–582.00)	1.00 (0.00–15.50)	0.001 **
	Placebo	394.00 (313.50–547.50)	328.00 (302.50–519.00)	0.334
CH_3_SH (ppb)	CPC	34.00 (22.00–53.00)	0.00 (0.00–2.00)	0.001 **
	Placebo	56.00 (27.00–75.00)	35.00 (24.00–53.00)	0.105
(CH_3_)_2_S (ppb)	CPC	3.00 (1.00–13.50)	2.00 (1.50–4.50)	0.221
	Placebo	4.00 (0.00–9.00)	2.00 (0.50–6.00)	0.529
Total VSCs (ppb)	CPC	446.00 (248.00–623.00)	6.00 (2.50–20.50)	0.001 **
	Placebo	468.00 (397.00–577.00)	389.00 (318.00–542.00)	0.105
PI	CPC	16.00 (7.55–18.25)	5.50 (2.90–6.75)	0.001 **
	Placebo	11.30 (7.75–16.65)	7.00 (5.90–9.40)	0.004 **
GI	CPC	9.00 (4.50–10.50)	3.00 (2.40–6.40)	0.012 **
	Placebo	6.80 (4.25–14.15)	5.30 (4.25–7.05)	0.086
TCI	CPC	0.50 (0.30–0.70)	0.20 (0.20–0.30)	0.003 **
	Placebo	0.60 (0.45–0.85)	0.60 (0.50–0.65)	0.188
Unstimulated Saliva (mL)	CPC	0.20 (0.20–0.40)	0.40 (0.25–0.50)	0.107
	Placebo	0.20 (0.15–0.55)	0.30 (0.15–0.70)	0.058

Abbreviations: H_2_S = hydrogen sulfide; CH_3_SH = methyl mercaptan; (CH_3_)_2_S = dimethyl sulfide; VSC = volatile sulfur compounds; PI = Plaque Index; GI = Gingival Index; TCI = Tongue Coating Index. Statistical significance: *p* < 0.01 (**).

**Table 3:** Species-level effect of the CPC mouthwash on the oral microbiome.

(A) CPC Mouthwash Group at Species Level							
Species (0.1% or More Reduction)	Baseline (%)	After 1 Month (%)	Difference (%)	Species (0.1% or More Increase)	Baseline (%)	After 1 Month (%)	Difference (%)
*Prevotella melaninogenica*	11.67	6.89	−4.78	*Prevotella scopos*	0.09	0.23	0.13
*Actinomyces sp.*	1.91	0.27	−1.64	*Neisseria oralis*	0.10	0.26	0.16
*Prevotella histicola*	2.71	1.16	−1.55	*Streptococcus mitis*	0.26	0.47	0.21
*Actinomyces gerencseriae*	1.36	0.02	−1.34	*Haemophilus sputorum*	0.02	0.23	0.21
*Actinomyces viscosus*	1.32	0.08	−1.24	*Actinomyces meyeri*	0.56	0.78	0.22
*Corynebacterium matruchotii*	1.16	0.01	−1.15	*Fusobacterium nucleatum*	1.42	1.79	0.37
*Actinobaculum sp.*	1.08	0.00	−1.08	*Porphyromonas catoniae*	2.12	2.90	0.78
*Actinomyces naeslundii*	1.02	0.05	−0.97	*Gemella sanguinis*	0.62	1.54	0.92
*Prevotella denticola*	1.04	0.10	−0.93	*Granulicatella adiacens*	3.30	4.53	1.23
*Prevotella loescheii*	0.66	0.00	−0.65	*Streptococcus sobrinus*	0.09	1.42	1.33
*Porphyromonas endodontalis*	0.73	0.13	−0.60	*Rothia mucilaginosa*	6.40	7.93	1.53
*Prevotella salivae*	0.87	0.31	−0.56	*Streptococcus parasanguinis*	2.45	4.46	2.01
*Veillonella alcalescens*	1.07	0.51	−0.56	*Neisseria perflava*	2.42	4.93	2.51
*Streptococcus peroris*	0.71	0.22	−0.49	*Veillonella parvula*	3.78	6.35	2.57
*Streptococcus oralis*	1.41	0.96	−0.45	*Neisseria flavescens*	1.41	4.60	3.19
*Selenomonas noxia*	0.45	0.01	−0.45	*Haemophilus parainfluenzae*	1.77	5.28	3.50
*Actinomyces odontolyticus*	2.97	2.52	−0.44	*Streptococcus salivarius*	7.72	12.23	4.51
*Prevotella oulorum*	0.46	0.03	−0.43	*Streptococcus infantis*	1.82	7.71	5.89
*Streptococcus sanguinis*	0.49	0.06	−0.42				
*Propionibacterium propionicum*	0.42	0.00	−0.42				
*Prevotella copri*	1.13	0.75	−0.38				
*Selenomonas genomosp.*	0.38	0.01	−0.37				
*Schlegelella thermodepolymerans*	0.45	0.08	−0.36				
*Leptotrichia wadei*	0.59	0.23	−0.35				
*Alloprevotella rava*	0.36	0.06	−0.30				
*Streptococcus tigurinus*	0.39	0.08	−0.30				
*Prevotella pallens*	1.39	1.10	−0.30				
*Rothia dentocariosa*	0.62	0.35	−0.28				
*Actinomyces massiliensis*	0.26	0.00	−0.26				
*Actinomyces oris*	0.27	0.01	−0.26				
*Prevotella nigrescens*	0.28	0.03	−0.26				
*Streptococcus anginosus*	0.27	0.03	−0.24				
*Streptococcus cristatus*	0.43	0.21	−0.22				
*Rothia aeria*	0.25	0.04	−0.21				
*Leptotrichia sp.*	0.24	0.05	−0.20				
*Prevotella sp.*	0.21	0.01	−0.20				
*Treponema medium*	0.19	0.00	−0.19				
*Capnocytophaga ochracea*	0.19	0.00	−0.19				
*Streptococcus australis*	0.66	0.48	−0.18				
*Selenomonas sputigena*	0.19	0.01	−0.17				
*Prevotella saccharolytica*	0.17	0.00	−0.17				
*Tannerella sp.*	0.16	0.00	−0.16				
*Neisseria bacilliformis*	0.16	0.01	−0.16				
*Selenomonas infelix*	0.16	0.01	−0.15				
*Leptotrichia shahii*	0.15	0.01	−0.14				
*Selenomonas artemidis*	0.15	0.00	−0.14				
*Gemella morbillorum*	0.13	0.00	−0.13				
*Actinomyces dentalis*	0.12	0.00	−0.12				
*Streptococcus sp.*	0.13	0.01	−0.12				
*Eikenella corrodens*	0.13	0.01	−0.12				
*Campylobacter gracilis*	0.12	0.01	−0.12				
*Treponema socranskii*	0.12	0.00	−0.12				
*Parvimonas micra*	0.12	0.00	−0.12				
*Leptotrichia buccalis*	0.11	0.00	−0.11				
*Lachnoanaerobaculum saburreum*	0.12	0.01	−0.11				
*Prevotella oris*	0.12	0.02	−0.11				
**(B) Placebo Mouthwash Group at the Species Level**							
**Species (0.1% or More Reduction)**	**Baseline** **(%)**	**After 1 Month** **(%)**	**Difference** **(%)**	**Species (0.1% or More Increase)**	**Baseline** **(%)**	**After 1 Month** **(%)**	**Difference** **(%)**
*Prevotella copri*	3.36	0.92	−2.43	*Actinomyces oris*	0.03	0.14	0.11
*Streptococcus anginosus*	1.99	0.21	−1.78	*Lachnoanaerobaculum orale*	0.12	0.24	0.12
*Prevotella nigrescens*	1.31	0.09	−1.22	*Neisseria flavescens*	0.14	0.26	0.12
*Corynebacterium matruchotii*	2.09	0.89	−1.20	*Streptococcus oralis*	0.56	0.70	0.14
*Actinomyces meyeri*	1.40	0.24	−1.16	*Haemophilus parainfluenzae*	2.00	2.14	0.15
*Porphyromonas catoniae*	4.17	3.01	−1.16	*Fusobacterium nucleatum*	2.03	2.18	0.15
*Prevotella aurantiaca*	1.09	0.33	−0.76	*Neisseria elongata*	0.07	0.22	0.15
*Veillonella alcalescens*	1.89	1.21	−0.68	*Peptostreptococcus stomatis*	0.56	0.74	0.17
*Schlegelella thermodepolymerans*	0.79	0.11	−0.68	*Actinomyces viscosus*	0.14	0.35	0.21
*Prevotella loescheii*	0.98	0.32	−0.65	*Prevotella pallens*	0.74	0.95	0.21
*Leptotrichia wadei*	1.31	0.66	−0.65	*Parvimonas sp.*	0.20	0.43	0.24
*Streptococcus peroris*	1.24	0.66	−0.59	*Rothia dentocariosa*	0.35	0.61	0.27
*Prevotella nanceiensis*	1.42	0.92	−0.50	*Streptococcus mutans*	0.01	0.32	0.31
*Streptococcus gordonii*	0.64	0.16	−0.48	*Gemella sanguinis*	0.66	1.03	0.37
*Streptococcus cristatus*	0.76	0.36	−0.41	*Streptococcus australis*	0.55	1.08	0.53
*Prevotella denticola*	0.43	0.03	−0.40	*Actinomyces odontolyticus*	2.17	2.99	0.82
*Alloprevotella tannerae*	0.59	0.20	−0.39	*Rothia mucilaginosa*	4.26	5.18	0.91
*Actinobaculum sp.*	0.59	0.22	−0.37	*Granulicatella adiacens*	1.89	3.55	1.65
*Leptotrichia hofstadii*	0.53	0.20	−0.33	*Streptococcus infantis*	1.11	2.82	1.71
*Streptococcus vestibularis*	0.30	0.00	−0.30	*Streptococcus parasanguinis*	2.02	4.23	2.20
*Actinomyces massiliensis*	0.32	0.05	−0.27	*Neisseria perflava*	3.40	5.72	2.32
*Prevotella oulorum*	0.38	0.11	−0.27	*Veillonella parvula*	3.69	6.19	2.50
*Rothia aeria*	0.34	0.08	−0.26	*Actinomyces graevenitzii*	1.50	5.61	4.10
*Prevotella oris*	0.30	0.04	−0.26	*Streptococcus salivarius*	4.98	10.21	5.23
*Streptococcus sanguinis*	0.45	0.20	−0.25				
*Neisseria oralis*	0.33	0.08	−0.25				
*Prevotella maculosa*	0.30	0.07	−0.23				
*Leptotrichia hongkongensis*	0.25	0.03	−0.22				
*Gemella morbillorum*	0.33	0.11	−0.22				
*Streptococcus lactarius*	0.39	0.20	−0.19				
*Prevotella melaninogenica*	10.62	10.43	−0.19				
*Selenomonas noxia*	0.41	0.22	−0.18				
*Actinomyces naeslundii*	0.45	0.28	−0.17				
*Lautropia mirabilis*	0.22	0.05	−0.17				
*Prevotella shahii*	0.31	0.14	−0.16				
*Prevotella micans*	0.20	0.04	−0.16				
*Actinomyces dentalis*	0.18	0.04	−0.14				
*Prevotella saccharolytica*	0.25	0.11	−0.14				
*Actinomyces sp.*	0.70	0.56	−0.14				
*Eubacterium brachy*	0.15	0.02	−0.13				
*Kingella oralis*	0.14	0.01	−0.13				
*Treponema medium*	0.18	0.05	−0.13				
*Capnocytophaga sputigena*	0.20	0.08	−0.13				
*Leptotrichia sp.*	0.16	0.05	−0.11				

## Data Availability

All of the clinical data and microbiome data are available in the Supplementary Materials.

## Supplementary Materials

The following supporting information can be downloaded at: https://www.mdpi.com/article/10.3390/jcm14134576/s1, Figure S1: Flowchart showing recruitment of patients, allocation, and dropout; Table S1: Results of tests on the normality of the data. S1 File: All the data analyzed in this study.
