# Supplementary material for: Effects of 0.05% Cetylpyridinium Chloride Mouthwash on Halitosis and Tongue Microbiota in Patients Undergoing Orthodontic Treatment: A Double-Blind Randomized Clinical Trial
Source: J Clin Med. 2025 Jun 27;14(13):4576. doi: 10.3390/jcm14134576 (PMC12249613; doi:10.3390/jcm14134576)
Supplement: Supplementary file 1 [file jcm-14-04576-s001.zip › Supplemental materials(Revise)/Supplemental.pptx]

## Slide 1
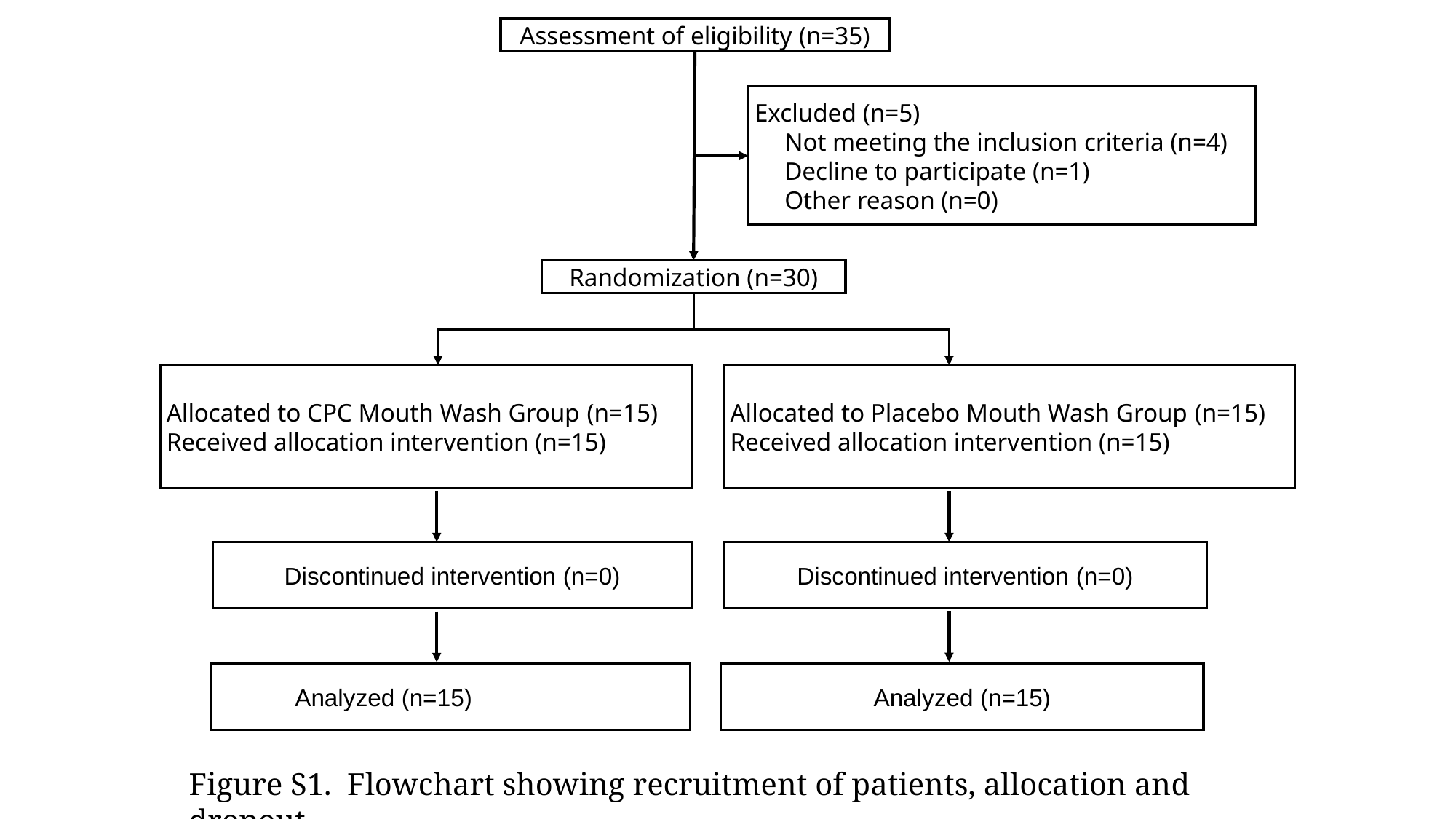

Assessment of eligibility (n=35)
Excluded (n=5)
　Not meeting the inclusion criteria (n=4)
　Decline to participate (n=1)
　Other reason (n=0)
Randomization (n=30)
Allocated to CPC Mouth Wash Group (n=15)
Received allocation intervention (n=15)
Allocated to Placebo Mouth Wash Group (n=15)
Received allocation intervention (n=15)
Discontinued intervention (n=0)
Discontinued intervention (n=0)
Analyzed (n=15)
Analyzed (n=15)
Figure S1. Flowchart showing recruitment of patients, allocation and dropout

## Slide 2
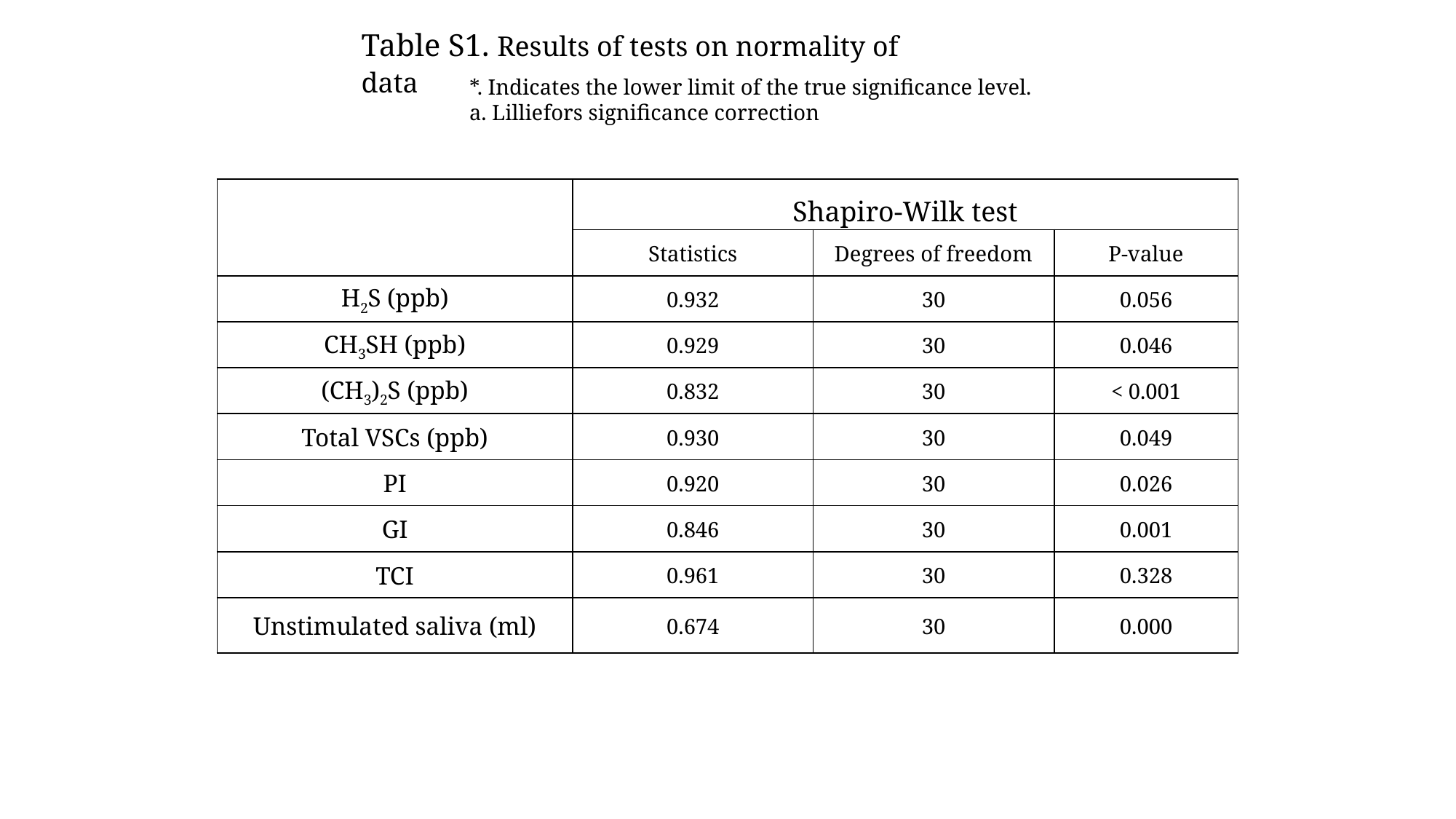

Table S1. Results of tests on normality of data
*. Indicates the lower limit of the true significance level.
a. Lilliefors significance correction
| | Shapiro-Wilk test | | |
| --- | --- | --- | --- |
| | Statistics | Degrees of freedom | P-value |
| H2S (ppb) | 0.932 | 30 | 0.056 |
| CH3SH (ppb) | 0.929 | 30 | 0.046 |
| (CH3)2S (ppb) | 0.832 | 30 | < 0.001 |
| Total VSCs (ppb) | 0.930 | 30 | 0.049 |
| PI | 0.920 | 30 | 0.026 |
| GI | 0.846 | 30 | 0.001 |
| TCI | 0.961 | 30 | 0.328 |
| Unstimulated saliva (ml) | 0.674 | 30 | 0.000 |
